# SGLT2 inhibitors as potentially helpful drugs in PI3K inhibitor-induced diabetes: a case report

**DOI:** 10.1186/s40842-021-00125-8

**Published:** 2021-07-20

**Authors:** Nicolas Sahakian, Lauranne Cattieuw, Clotilde Ramillon-Cury, Audrey Bégu-Le Corroller, Pascale Silvestre-Aillaud, Sophie Béliard, René Valéro

**Affiliations:** 1 Department of Nutrition, Metabolic Diseases and Endocrinology, University Hospital La Conception, 147 boulevard Baille, 13005 Marseille, France; 2 Aix Marseille Univ, APHM, INSERM, INRAE, C2VN, 27 boulevard Jean Moulin, 13005 Marseille, France; 3 Service de Nutrition, Maladies Métaboliques Et Endocrinologie, Centre Hospitalo-Universitaire de La Conception, 147 Boulevard Baille, 13005 Marseille, France

**Keywords:** Cancer treatment, Case report, Diabetes, Hyperglycemia, Insulin resistance, Metabolic side effects, PI3K, PI3K inhibitor, SGLT2 inhibitor

## Abstract

**Background:**

Hyperglycemia is the most common side-effect of phosphatidylinositol 3-kinase (PI3K) inhibitors that are approved for the treatment of some advanced or metastatic breast cancers. This side-effect is likely due to the central role of PI3K in insulin signalling. Here we report the use of a sodium-glucose cotransporter 2 (SGLT2) inhibitor to manage severe hyperglycemia.

**Case presentation:**

We describe a 74-year-old woman who developed severe uncontrolled hyperglycemia after commencing alpelisib, a new oral PI3K inhibitor indicated for a metastatic breast cancer, despite taking oral anti-diabetic drugs, metformin and vildagliptin, combined with intravenous insulin infusion of up to 250 units/day. The introduction of the SGLT2 inhibitor dapagliflozin rapidly improved blood glucose with a drastic reduction in insulin dosage, from 250 to 12 units/day, and without significant side-effects.

**Conclusions:**

We report the successful management of hyperglycemia induced by alpelisib using a SGLT2 inhibitor without the need to discontinue effective cancer treatment.

## Background

Alpelisib is a new oral α-specific inhibitor of class I phosphatidylinositol 3-kinase (PI3K) [1] approved in several countries, in combination with fulvestrant, for the treatment of postmenopausal women with hormone receptor (HR)-positive, human epidermal growth factor receptor-2 (HER2)-negative, PIK3CA-mutated, advanced or metastatic breast cancer. SOLAR-1, a phase 3 trial, reported prolonged progression-free survival with alpelisib-fulvestrant compared to placebo-fulvestrant treatment in patients with PIK3CA mutations [2]. Due to the central role of PI3Kα in regulating glucose metabolism [3], particularly insulin signalling, previously used PI3K inhibitors such as idelisib and copanlisib have already been associated with hyperglycemia [4]. In the SOLAR-1 trial, hyperglycemia was the most commonly reported adverse event with 63.7% vs 9.8% reporting any grade hyperglycemia, 36.6% vs 0.7% grade 3 (blood glucose > 250–500 mg/dL) and grade 4 (blood glucose > 500 mg/dL) hyperglycemia, respectively, comparing alpelisib-fulvestrant to placebo-fulvestrant treatment. This led to cessation of alpelisib in 6.3% of patients vs 0% in the placebo group [2]. Recommendations for managing hyperglycemia indicate dose adjustment or interruption of alpelisib and initiation or intensification of anti-diabetic treatment. The preferred option for treating alpelisib-induced hyperglycemia is metformin. Thiazolidinediones or dipeptidyl peptidase IV (DPPIV) inhibitors may be added or used in case of metformin intolerance. Insulin may be used until hyperglycemia resolves, usually in 1–2 days due to the short half-life of alpelisib [2, 5].

Here, we report a case of successful management of severe hyperglycemia induced by alpelisib, using a sodium-glucose cotransporter 2 (SGLT2) inhibitor, dapagliflozin, without the need for discontinuing alpelisib treatment.

## Case presentation

A 74-year-old woman was diagnosed in 2011 with HR-positive, HER2-negative, PI3KCA-mutated breast cancer, initially treated by surgery, radiotherapy, chemotherapy, hormonotherapy, then hormonotherapy plus the protein kinase inhibitor palbociclib. Her breast cancer progressed, with bone metastases in 2015 and liver metastases in 2019. In accordance with recommendations, treatment with alpelisib plus fulvestrant was initiated. Alpelisib was introduced at a dose of 300 mg/day. She had no history of diabetes. Fasting blood glucose and HbA1c were respectively 104 mg/dL and 5.9% (41 mmol/mol) before the initiation of alpelisib.

A few weeks after commencing alpelisib, she developed severe hyperglycemia up to 300 mg/dL with the loss of 4 kg or 6% body weight, but without ketosis. Following management guidelines, treatment with metformin (1000 mg twice daily) and a DPPIV inhibitor vildagliptin (50 mg twice daily) was started, subsequently subcutaneous and intravenous insulin therapy was added, with insulin doses up to 150 units/day. Finally, she was referred to our tertiary diabetes department due to persistent poor glycemic control despite these treatments including intravenous insulin therapy. Her weight at entry was 64 kg, body mass index (BMI) 26.3 kg/m^2^ and HbA1c was 9.2% (77 mmol/mol). Etiological assessment of diabetes identified no cause other than iatrogenic diabetes due to alpelisib. Anti-GAD (glutamic acid decarboxylase), anti-IA2 (tyrosine phosphatase 2) and ZnT8 (Zinc transporter 8) antibodies were negative and pancreatic CT scan showed no abnormality. Fasting plasma C-peptide concentration was elevated (1.75 nmol/L).

Following published guidance for managing adverse events of alpelisib [6], the dose of alpelisib was reduced to 250 mg/day then 200 mg/day, treatment with metformin was increased to 1000 mg three times/day, vildagliptin was stopped and continuous intravenous insulin was increased to 250 units/day. Despite optimized insulin treatment combined with oral anti-diabetic drugs, severe hyperglycemia (from 200 to 300 mg/dL) persisted.

Before stopping alpelisib as recommended [6], since it showed anti-cancer efficacy, we decided to introduce the SGLT2 inhibitor dapagliflozin at a dose of 10 mg/day. The patient had no countraindication to dapagliflozin, particularly no renal failure. Introduction of dapagliflozin improved glycemic control within 3 days (blood glucose levels 100—150 mg/dL), with a decreased intravenous insulin dose from 250 to 100 units/day.

**Fig. 1:**
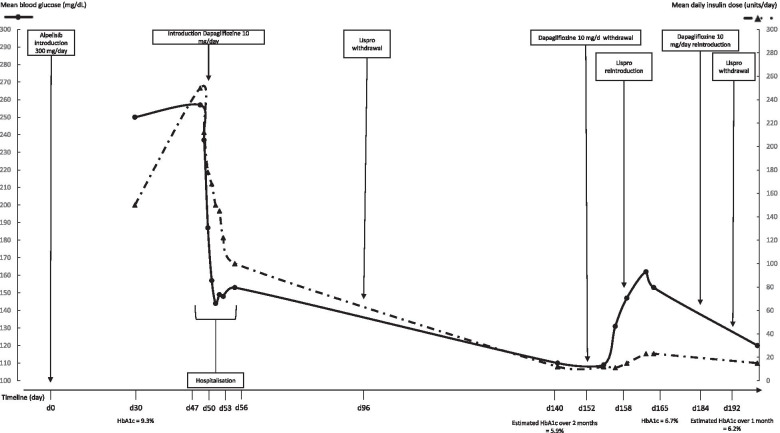
Evolution of blood glucose and anti-diabetic treatments after alpelisib introduction

Good treatment tolerance was observed over one week, with no weight loss or other side-effects. Alpelisib was thus maintained at a dose of 200 mg/day. Treatment after discharge from hospital was metformin (1000 mg three times/day), dapagliflozin (10 mg/day) and subcutaneous insulin (lispro 48 units/day and glargine 50 units/day). After discharge, glycemic control continued to improve allowing discontinuation of fast-acting insulin (lispro) and a drastic reduction in dose of long-acting insulin (glargine) from 50 to 12 units/day. Three months after discharge the estimated HbA1c (flash glucose monitoring) over 2 months was 5.9% (41 mmol/mol). However, in view of 3 kg or 5% body weight loss, the oncologist elected to stop dapagliflozin, necessitating reintroduction of subcutaneous lispro at up to 10 units/day combined with glargine (13 units/day), in view of the deterioration in glycemic control (mean blood glucose increased from 109 mg/dL to 163 mg/dL with blood glucose up to 262 mg/dL). One month after dapagliflozin withdrawal HbA1c was 6.7% (50 mmol/mol). Fasting C-peptide levels remained elevated at 1.42 nmol/L. Dapagliflozin was reintroduced at a dose of 10 mg/day, giving good glycemic control after one week (blood glucose approximately 120 mg/dL and estimated HbA1c (flash glucose monitoring) over 1 month 6.2% (44 mmol/mol)). Subcutaneous Lispro was again discontinued and alpelisib was maintained at a dose of 200 mg/day (Fig. 1). The regular monitoring of ketone capillary blood measurement (beta-hydroxybutyrate) using the Optium FreeStyle glucose/ketone system was normal (< 0.5 mmol/L) during the follow-up, both before and after the start of dapagliflozin.

## Discussion

New anti-cancer drugs have become available recently, with efficacy in treating cancer but with the challenge of managing side effects such as hyperglycemia. Indeed, severe hyperglycemia can lead to life-threatening acute complications of diabetes including ketoacidosis or hyperosmolar coma, leading to dose adjustment or permanent withdrawal of the drug, even where treatment is effective in controlling cancer evolution, potentially impacting patient life expectancy. Hyperglycemia is the most common side-effect in patients treated with PI3K inhibitors, including alpelisib. Although age is an important risk factor for the development of iatrogenic diabetes, a study has shown that age was not a predictor for developing diabetes in patients treated with PI3K inhibitors but that a younger age predicts diabetes remission after drug discontinuation [7]. The PI3Kα pathway is involved in glucose metabolism via insulin receptor signalling, and in glucose uptake and glycogen synthesis. Its inhibition can contribute to glucose intolerance and diabetes via insulin resistance, resulting in impaired glucose uptake in muscle and increased hepatic gluconeogenesis [8]. Class IA PI3K may also contribute to regulation of insulin secretion [9]. Our case report favors an insulin-resistance mechanism to explain hyperglycemia, with an increase in fasting C-peptide concentration and an insulin dose of up to 3.9 units/kg required to control diabetes. We did not find any other major factor, apart from alpelisib, that could explain this extreme insulin resistance. In particular, she did not take corticosteroid, her oral food intake varied from 1200 to 1700 kcal/day, the amount of glucose infused during the intravenous insulin therapy was 100 g/day (400 kcal/day) and the C-reactive protein level was slightly elevated (10.1 mg/L). Recommendations for management of hyperglycemia-related to alpelisib treatment include commencing insulin-sensitizing oral antidiabetic treatment, such as metformin and pioglitazone, with the option of insulin as rescue medication [6]. This strategy failed in our patient and the treatment was unable to counteract insulin resistance induced by alpelisib. Before stopping alpelisib treatment, which showed good anti-tumor efficacy, we decided to use the most recent class of antidiabetic drug (SGLT2 inhibitors) which acts independently of insulin signalling. These drugs inhibit glucose reabsorption in renal proximal tubules, increasing urinary glucose excretion and reducing circulating glucose [10]. This therapeutic class requires sensitization and patients need to be informed of the risk of side-effects including: hypoglycemia in patients receiving insulin or sulfonylurea, genital infections and very rare cases of Fournier’s gangrene, volume depletion (hypotension, hypovolaemia and dehydration), rare cases of hyperglycemic or euglycemic ketoacidosis [11, 12] and the necessity to stop treatment prior to any surgery. Indeed, a previous case report described a euglycemic ketoacidosis within one week after the introduction of the canagliflozin to treat a diabetes induced by another PI3K inhibitor (taselisib) [13]. Predisposing and precipitating factors of diabetic ketoacidosis during SGLT2 inhibitor therapy are still incompletely understood but several precipitating factors are now well identified such as: surgical stress, limited perioperative withholding of SGLT2 inhibitor, insufficient pre- and postoperative or reduced dose of insulin, reduced perioperative oral intake, preoperative low carbohydrate diets, concurrent illness (infection, malignancy, dehydration and decreased oral intake), alcohol use and recent initiation of SGLT2 inhibitor therapy [14]. The comparison of the two case reports is complex because the patients were not treated by the same PI3K inhibitor and by the same anti-diabetic drugs. However, we can identify at least 4 precipitating factors of diabetic ketoacidosis in the previous case report, that are absent in our case report, such as: decreased oral intake, dehydration, no insulin treatment and oral corticosteroid treatment. Despite the remarkable efficacy of dapagliflozin, the oncologist stopped this treatment after 3 kg of weight loss, even though it was difficult to determine if the advanced cancer or side-effects of the medications used, including alpelisib, were the cause. In two recent studies, mean weight loss under dapagliflozin was between 2.2 and 3.2 kg [15] and between -3.31 and -4.06% from baseline [16].

## Conclusion

Here we report the successful use of dapagliflozin (an SGLT2 inhibitor) to manage severe hyperglycemia induced by alpelisib (a PI3K inhibitor) which might then be continued in view of its good anti-cancer efficacy. Longer-term larger studies are required to confirm the usefulness of SGLT2 inhibitors in the management of PI3K inhibitor-induced hyperglycemia and to monitor for serious side-effect.

## Data Availability

The data used in this case report are available in the patient’s medical record and can be disclosed by the corresponding author on reasonable request.

## Abbreviations

BMI: Body mass index
DPPIV: Dipeptidyl peptidase IV
GAD: Glutamic acid decarboxylase
HER2: Human epidermal growth factor receptor-2
HR: Hormone receptor
IA2: Tyrosine phosphatase
PI3K: Phosphatidylinositol 3-kinase
SGLT2: Sodium-glucose cotransporter 2
ZnT8: Zinc transporter 8
